# Dielectric Permittivity in Copper Leaching: A Review

**DOI:** 10.3390/s25030794

**Published:** 2025-01-28

**Authors:** Marcos Andreu, Robert Zwick, Moe Momayez

**Affiliations:** 1 Department of Mining and Geological Engineering, The University of Arizona, Tucson, AZ 85719, USA; mmomayez@arizona.edu; 2 Freeport McMoRan, Phoenix, AZ 85004, USA; rzwick@fmi.com

**Keywords:** dielectric permittivity, copper leaching, ore permittivity

## Abstract

The leaching process for copper extraction has garnered significant attention due to its critical role in meeting the rising demand for copper, driven by global trends towards decarbonization and electrification. The accurate measurement of variables is essential for process control, prompting the development of advanced sensor technologies. This paper reviews the applications of dielectric permittivity measurements in the mining industry, focusing on their potential to enhance the monitoring and optimization of copper leaching processes. It evaluates the suitability of permittivity-based sensors, analyzing their advantages and limitations, and discusses the implications for process control and economic optimization. The study highlights the integration of permittivity measurements into existing monitoring systems, aiming to improve efficiency, reduce environmental impact, and increase ore recovery rates. This comprehensive review provides insights into the current state of permittivity measurement technologies and their future prospects in the context of copper leaching.

## 1. Introduction

**Figure 1:**
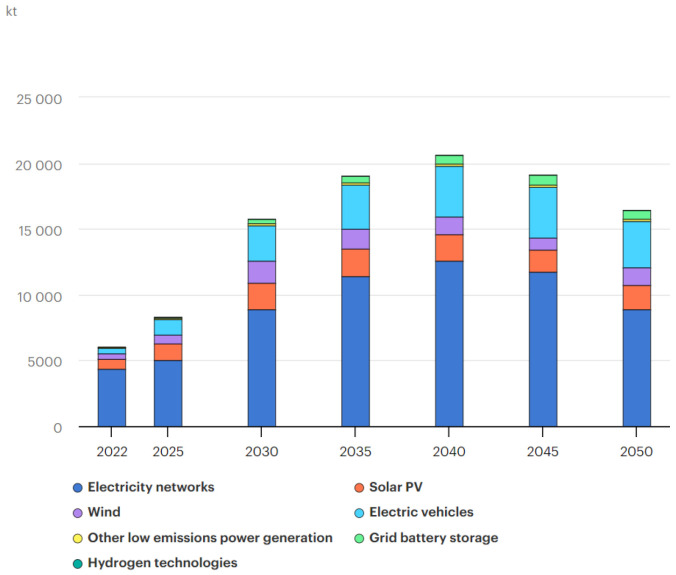
IEA 2023: copper demand for clean energy in the Net Zero Emissions by 2050 scenario [3].

The growing global trend towards the decarbonization and electrification of society implies an increasing and pressing demand for strategic minerals. Among these, copper stands as a fundamental and indispensable cornerstone in the electrification process. It plays a pivotal role in the green technology scenario, where the predicted copper consumption is expected to be 263 kg per person by 2070 in the US, as proposed by Elshkaki [1] and more recently by He [2]. The demand for copper in the scenario of Net Zero Emission by 2050, as outlined by the International Energy Agency (IEA), also supports a higher consumption forecast, as depicted in Figure 1. In this figure, we can observe that the highest demand is expected to be reached during the green transition phase (up until 2040). During this phase, the expansion of various sectors will require significant quantities of copper, particularly in the power transmission and electromobility sectors [3].

To meet the rising demand for copper and address the challenge of decreasing ore grades, there is value in exploring and implementing technological advancements in copper extraction methods. These play a crucial role in bridging the gap between the supply and demand for copper. Hydrometallurgical techniques have gained considerable attention in the United States. For instance, copper recovered in concentrates and precipitates made up 53% of the total mine output, which represented a decline of 12%, dropping to 643,000 tons in 2020 from 730,000 tons in 2019. On the other hand, copper produced through solvent extraction and electrowinning (SX–EW) accounted for 47% of mine production, with a 6% increase, reaching 559,000 tons compared to 527,000 tons during the previous year. These reflect the mining industry’s ongoing commitment to advancing research and development in leaching technology. Similarly, the Chilean National Copper Corporation (CODELCO) promotes innovative research in hydrometallurgical processes through programs such as “Piensa Minería y Desafíos Codelco 2023” [4,5].

### Significance of Monitoring and Advancements in Technology

**Figure 2:**
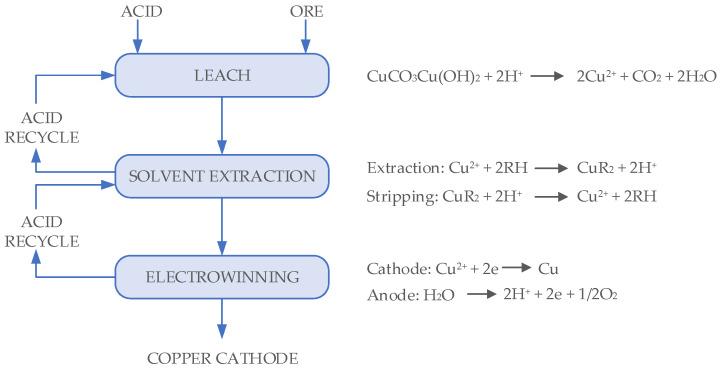
Copper heap leaching flow diagram and reactions. Source: Modified from Schlesinger et al. [8].

The principles and mechanisms of leaching copper-bearing oxide minerals are well understood, as illustrated in Figure 2, and recovery models and reactions are thoroughly described in the literature [6,7,8]. Theoretical models can be enhanced using measurements complemented by numerical approaches, as shown in [9,10]. The proposed techniques assist the development of models that allow the prediction of the behavior of the heap leaching process, looking for the potential optimization of manipulated variables and assisting in the decision-making process. In a similar way, close-loop control systems hold the potential to significantly enhance copper recovery while concurrently reducing costs and losses [11]. Authors involved in the industry have proposed a prototype of intelligent recommendation systems that support decision-making for the selection of the modes of operations in heap copper leaching. These systems aim to reduce the effort required in generating information, analysis, and design for planning the modes of operations, which is crucial for profitability in copper mining [12].

The integration of process automation is deeply integrated into pyrometallurgical copper extraction, providing optimization and control of the various processes involved. This inclusion of control systems in these processes owes its success to a number of sensors and instruments that facilitate the measurement of critical variables [13,14]. Heap leaching, on the other hand, not only presents geotechnical challenges, but innovations in sensors and monitoring are also key for the future optimization of production [15,16]. Recently, there has been a surge of innovation in artificial intelligence (AI) and machine learning (ML) techniques for the study of parameters, identification, and modelling of extractive metallurgy [17,18,19]. This has given rise to soft sensors that essentially allow the estimation of a target variable based on the measurements of available sensor data, trying to bridge the gap in monitoring technology and showing promising results [20,21,22]. However, the effectiveness of sensors based on physical and chemical principles for monitoring various parameters such as leach solution flow rates, temperatures, and pH levels among others are preferred.

The purpose of this paper is to explore the applications of sensors based on electromagnetic properties, specifically permittivity, as alternatives for monitoring the copper leaching process. In this context, permittivity measurements could be used to monitor changes in dielectric properties, which can indicate alterations in moisture content, mineral composition, and other factors critical to the efficiency of the leaching process. The document is structured around the following specific objectives: (*i*) providing an overview of the existing permittivity measurement techniques employed in the mining industry, with an emphasis on their principles, advantages, and limitations; (*ii*) assessing the suitability of permittivity measurements for monitoring copper leaching, including a discussion on how these measurements can be integrated into the process; (*iii*) highlighting the advantages and limitations of using permittivity measurements in copper leaching, addressing issues such as accuracy, cost-effectiveness, and scalability; and (*iv*) discussing the potential impact of using permittivity measurements on the mining industry, including considerations related to enhanced efficiency, reduced environmental impact, and improved ore recovery rates. This structured approach will allow for a clear and systematic examination of permittivity measurements and their role in copper leaching monitoring.

## 2. Dielectric Permittivity

### 2.1. Definition and Principles of Dielectric Permittivity

**Figure 3:**
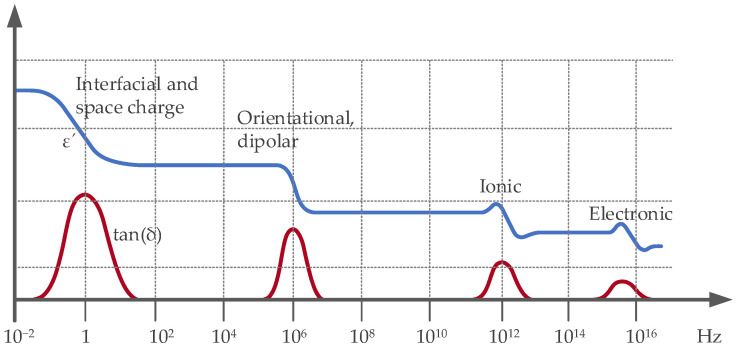
Dielectric permittivity (blue) and loss tangent (red) in wide frequency ranges. Source: Modified from Keysight Technologies (Santa Rosa, CA, USA) [23].

When exposed to an external electric field, materials undergo polarization—a reorganization of their molecules. This polarization effect can occur at various levels across different frequency ranges, as shown in Figure 3 [23]. For instance, electrode polarization occurs when free ions in a solution remain close to the electrodes of the electromagnetic wave source, typically manifesting at frequencies below 10 kHz. Spatial polarization involves the displacement of clusters of ions in solution and typically occurs in the range of 10 kHz to 10 MHz. In contrast, orientational polarization tends to occur between the frequency range of 1 MHz to 1 GHz. Moreover, dielectric polarization arises in materials with bound charges, such as insulators and certain types of capacitors. It involves the alignment of dipoles within the material in response to an external electric field, commonly manifesting in frequency ranges above 1 GHz. Dielectric polarization plays a critical role in the operation of capacitors and dielectric materials used across a spectrum of electronic devices and systems [24]. The degree of polarization directly correlates with the material’s ability to store energy within an electric field; this property is quantified by the dielectric permittivity, also referred to as the dielectric constant in this paper [24,25,26]. Early studies focused on the dispersion and absorption of dielectric materials, such as polar liquids and solutions, and then extended to polar solids and their dependence on the frequency of their complex permittivity [27].

Essentially, dielectric permittivity serves as a crucial parameter, representing a fundamental property of a medium. Its significance lies in influencing the propagation of the electric field within the medium, making it a relevant factor in understanding and characterizing the behavior of minerals under such conditions. In Sihvola [24], the authors define the relationship between the electric field vector (***E***) and the electric flux density vector (***D***) in a medium:

(1) D=εE,

where ε denotes the absolute permittivity in tensor form, which can be reduced to a scalar representation on isotropic materials, measured in F/m. The permittivity of a given material is usually represented relative to that of the free space ($\varepsilon_{0}\approx 8.8542\times 10^{-12}\;F/m$).

(2) εr=εε0,

Also known as the dielectric constant, the magnitude of this dimensionless parameter denotes the material’s dielectric nature. For instance, air has a relative permittivity of 1, while water approaches 80. It is worth mentioning that minerals and materials involved in the copper extraction process have dielectric constants within this range. Some common values for substances involved in copper hydrometallurgy are shown in Table 1 [28,29].

### 2.2. Complex Permittivity

The expression for the electric flux density in (1) assumes a lossless medium. However, certain materials convert the stored energy in electric fields into current and, consequently, heat. These types of media are referred to as “*Lossy*” [30]. In such cases, the permittivity is expressed as a complex number:

(3) $$\varepsilon =\varepsilon^{\prime }-j\varepsilon^{\prime \prime },$$

where the real part $\varepsilon^{\prime }$ relates to physical permittivity and is usually referred to as a dielectric constant, and the imaginary part $\varepsilon^{\prime \prime }$ is related to the energy lost as heat as a function of its conductivity *σ*. Thus, we can define the following:

(4) $$\varepsilon^{\prime }=\varepsilon ,\;\varepsilon^{\prime \prime }=\frac{\sigma }{\omega }.$$

where *ω* is the angular frequency of the field in rad/s [31]. The measure of the efficiency with which a material converts electromagnetic energy into heat the loss tangent is defined as the ratio of the imaginary part of the permittivity to its real part. Mathematically, it is expressed as follows:

(5) $$\tan\delta =\frac{\varepsilon^{\prime \prime }}{\varepsilon^{\prime }}=\frac{\sigma }{\omega \varepsilon^{\prime }}\;,$$

A higher loss tangent indicates a material’s greater efficiency in converting electromagnetic energy into heat, reflecting its dissipative nature. Conversely, a lower loss tangent suggests reduced losses and a material’s better ability to store energy in the electric field with minimal dissipation [32]. We can recognize that permittivity theory is strongly linked to the development of electromagnetic theory and its practical use is dedicated to the study of material properties. However, the measurement of permittivity is not solely dependent on the intrinsic properties of the material; it is also influenced by several external factors that can significantly impact its measurements.

### 2.3. Porosity and Moisture

**Figure 4:**
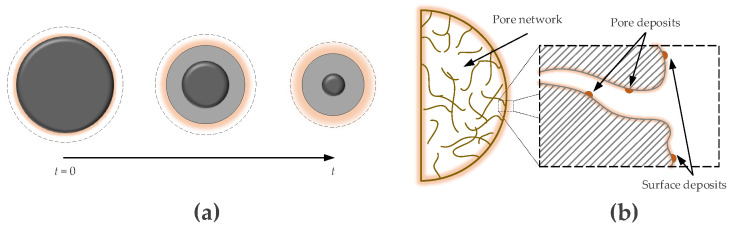
Leaching effects at ore particle levels. (**a**) Particles shrinking over time. (**b**) Intraparticle deposit dissolution. Source: Modified from Wen et al. [33].

During the leaching reaction, the larger particles shrink with the entrapment of valuable metal ions into a pregnant leach solution (PLS), as shown in Figure 4a [33]. The smallest particles are transported by the percolating solution towards the bottom of the pile, increasing the percentage of void space but at the same time increasing the permeability of the ore [34]. Similarly, when studying the effect of the leaching process on the residual content of test columns, researchers have found that the size distribution of the ore changes. Results from MRI scanning have confirmed this behavior where the smaller particles migrate to the bottom of the column. However, the dynamic behavior is not captured, and only the initial and final conditions of the leaching column are known [34,35].

While leaching, individual particles also undergo intra-particle diffusional processes, as shown in Figure 4b, meaning that the lixiviant not only dissolves valuable and gang minerals located on the particle’s surface but also internal deposits, thus increasing the porosity [36]. In fact, lixiviant concentrations are a key factor in changes in the void space with respect to ores with higher concentrations of H_2_SO_4_ (50 g/L) and Fe^3+^ (10 g/L), with void space changes of up to 17,800% in sulfide ores [37].

Alterations in soil porosity have a direct impact on dielectric permittivity. In fact, prior research has explored the estimation of porosity through the measurement of the dielectric constant in various mixtures of minerals, clay, non-clay solids, and solutions. The values estimated through this method have shown a strong correlation with their respective theoretical values [38]. Moreover, electrical spectroscopy theory has been developed for porous particles suspended in electrolytes [39,40].

**Figure 5:**
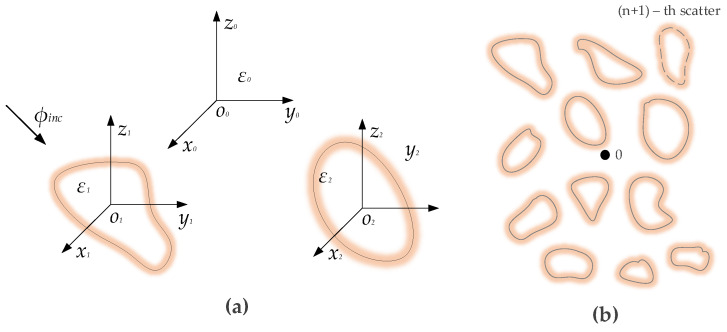
Inhomogeneously scattered media in dielectric materials. (**a**) Scattered media with different dielectric permittivity values and (**b**) scattered media. Source: Weng Cho Chew [43].

Early studies within the range of 0.1 to 100 MHz on saturated soil have established that the dielectric permittivity value is strongly influenced by moisture; they have also shown that porosity can be predicted with dielectric methods [41]. Electromagnetic techniques such as time-domain reflectometry (TDR) are recognized in the field of geotechnical engineering for assessing soil properties. This method measures the time it takes for an electromagnetic pulse to travel through a soil sample. By analyzing the travel time and waveform reflections, TDR can determine the soil’s permittivity, which is correlated with moisture content. Porosity, at the same time, can be estimated using information on water content and dielectric permittivity. These parameters are interlinked when studying the permittivity of uniform, porous geomaterials [42]:

(6) $$\sqrt{{\varepsilon^{\prime }}_{s}}=\varphi \sqrt{{\varepsilon^{\prime }}_{f}}+\left(1-\varphi \sqrt{\varepsilon_{m}^{\prime }}\right),$$

where $\varepsilon_{s}^{\prime }$ is the observed relative permittivity of the investigated sample, φ is the sample porosity, $\varepsilon_{f}^{\prime }$ is the relative permittivity of the fluid, and $\varepsilon_{m}^{\prime }$ is the relative permittivity of the geomaterial matrix. This concept is actively applied in geoscience and the study of inhomogeneous media, where special emphasis has been placed on the effect that layered and scattered media, as shown in Figure 5, that possess different dielectric properties and where the transmission and reflection of electromagnetic waves are impacted by these properties [43].

### 2.4. Ionic Concentration

The changes in the concentration of copper and iron ions in the PLS during the leaching period may influence measurements of the dielectric constant or permittivity. Early models, such as those by Hasted [44], on the dependence of the dielectric constant of the electrolyte solution as a function of the concentration of dissolved ions proposed a linear relationship as follows:

(7) $$\varepsilon =\varepsilon_{\omega }+2\bar{\delta }c,$$

where $\varepsilon_{\omega }$ is the dielectric constant of the solvent (water), *c* denotes the concentration of the solute (salt), and $\bar{\delta }$ denotes the adjusting parameter that considers the contribution of the ions being dissolved (Na^+^ and Cl^−^). The absorption coefficient method was implemented to determine the real and imaginary components of dielectric permittivity. However, the accuracy of the model is limited to concentrations lesser than 2 M. Most recently, Gavish [45], showed an improvement over this model considering micro-field theory, extending the range of concentrations up to 6 M and including the effect of temperature in the dielectric constant, as shown in their experimental results. The results of both papers show that an increase in the concentration of ions reduces the overall dielectric constant of the solution. Further studies of ion concentrations in aqueous solutions carried out by Liu [46] discuss how the dielectric constant responds to water changes with varying salt concentrations. This is modeled through a fourth-order *Poisson–Bikerman* equation, indicating that dielectric permittivity is influenced by the concentration of ions in the solution. The dielectric function produced by the model depends on both the charge density of ions and their concentrations. This means that as ion concentration changes, the dielectric constant of the solution also varies.

Research by Nguenouho [47] expands the scope to a more complex matrix of elements, including carbohydrates, in a ternary mixture that considers water, sodium chloride, and sucrose. The authors studied the changes in permittivity given variations in the concentration of these elements in water. The dielectric constant was measured using a broadband RF sensor operating in the microwave range. This was achieved by capturing scattering parameters (S-parameters), particularly the S_21_ parameter, which represents the ratio (gain) between the input and output voltages. The paper also provides a design for an RF antenna and emphasizes the opportunity for research into non-destructive methods, alongside mathematical models, that do not require the consumption of the sample to obtain accurate estimations or predictions of dielectric permittivity.

Dielectric permittivity is a parameter that not only varies widely from material to material but is also influenced by factors such as moisture, porosity, ionic concentration, and temperature. The study of the dielectric constant at a wide frequency range could provide an opportunity to improve the estimation of recovered copper minerals by measuring changes in ore permittivity while the leaching process is taking place at the heap.

## 3. Methods to Determine Permittivity

One of the most effective ways to measure permittivity is through electromagnetic methods. Electromagnetic waves travel and interact with the material to be studied, and these techniques are capable of capturing variations in dielectric properties. The main principles, techniques, and applications of electromagnetic methods in permittivity measurement are as follows.

*Time-domain reflectometry (TDR)*: This method involves sending an electromagnetic pulse along a probe inserted into the material. The pulse travels through the material and is reflected back when it encounters a change in electric properties (impedance), as could be the case for the end of the probe or a different material layer. The time it takes for the pulse to travel to the end of the probe and back is measured. This travel time is directly related to the permittivity of the material. Higher permittivity means the pulse travels slower, and lower permittivity means it travels faster. Relative permittivity (ε) can be calculated as follows:

(8) $$\varepsilon =\sqrt{\frac{ct}{2L}},$$

where *c* is the speed of light in a vacuum, using *t* as the travel time and *L* as the length of the probe. TDR is widely used in soil science to measure soil moisture content, as the permittivity of soil changes with its water content. It is also used in materials science to characterize dielectric films and other materials [48]. Newer research into the applications of and improvements in TDR has been carried out, such as in Wang [49], where the authors include the implementation of ML tools, specifically convolutional neural networks, to improve the interpretation of waveforms measured along the length of the probe relative to heterogeneous moisture in soil.

*Capacitance method*: These sensors consist of electrodes that are buried in soil, which form a capacitor, with the soil composition acting as the dielectric material. When an electric field is applied to the electrodes, the capacitance of the system changes based on the dielectric properties of the soil. As soil moisture levels vary, the dielectric constant of the soil changes, which in turn affects capacitance. The capacitance values are measured and analyzed to estimate the soil’s permittivity as follows:

(9) $$\varepsilon =\frac{Cd}{A},$$

where *C* is the measured capacitance, *A* is the area of the buried electrodes, and *d* is the distance between the electrodes [23]. Wu [50] researched the relationship between water contents and the dielectric constant using the electric capacitance method with parallel rod electrodes at 1 kHz. This relationship is found to be nonlinear, and several models were fitted to the data and compared. The results indicate that higher soil moisture levels draw higher dielectric constants, which leads to increased capacitance.

*Vector network analyzer with coaxial probe*: The coaxial probe is an open-ended sensor that is in contact with the material under test (MUT), introducing an electromagnetic field into the material. The vector network analyzer (VNA) measures the S-parameters, which describe how the electromagnetic waves are reflected and transmitted through the material. In a study by Šarolić [51], the authors determined the dielectric constant of saline solutions at various concentrations of NaCl using this method. Dielectric permittivity was calculated using the raw measurements of reflection coefficients and their postprocessing calculation; here, relative permittivity is calculated as follows:

(10) $$\varepsilon =\frac{A_{1}\Gamma -A_{2}}{A_{3}-\Gamma },$$

where Γ is the reflection coefficient of the MUT, while constants *A*_1_, *A*_2_, and *A*_3_ are unknowns determined by the calibration of the probe. The calibration is performed by measuring the reflection coefficient of the probe in open $\Gamma_{O}$, short-circuit $\Gamma_{S}$, and the reflection coefficient $\Gamma_{L}$ of a liquid with known permittivity $\varepsilon_{L}$. Then, the following is solved:

(11) $$A_{1}=\frac{\varepsilon_{L}\left(\Gamma_{S}-\Gamma_{L}\right)-\left(\Gamma_{S}-\Gamma_{O}\right)}{\left(\Gamma_{L}-\Gamma_{O}\right)},\;A_{1}=\frac{\varepsilon_{L}\Gamma_{O}\left(\Gamma_{S}-\Gamma_{L}\right)-\Gamma_{L}\left(\Gamma_{S}-\Gamma_{O}\right)}{\left(\Gamma_{L}-\Gamma_{O}\right)},\;A_{3}=\Gamma_{S}.$$

The results of the paper and the calculated permittivity, analyzed over a frequency range of 0.5 to 18 GHz, clearly show that this strategy is capable of capturing changes in the behavior of the solution, with lower relative permittivity and higher conductivity at increasing concentrations of salt.

*Resonant cavity method*: The resonator is typically a metallic enclosure with a specific shape (rectangular, cylindrical, or spherical) that supports standing waves at certain frequencies. When a material sample is introduced into the cavity, it interacts with the electromagnetic field inside the resonator. The presence of the material causes a shift in the resonant frequency and a change in the quality factor (Q-factor) of the cavity. These changes are directly related to the dielectric properties of the material. By measuring the shift in resonance frequency and the change in Q-factor, the permittivity of the material can be determined. This is typically carried out using a VNA to measure the resonant frequencies and their shifts accurately [52].

*Ground penetrating radar (GPR)*: These systems are well established, recognized, and used in geotechnical engineering to capture two-dimensional images of the subsurface. GPRs consist of two main components: (*i*) a transmitter antenna array and (*ii*) a receiver antenna array. The transmitter emits short pulses of electromagnetic waves into the ground. When these waves encounter materials with different dielectric properties, part of the energy is reflected back to the surface and is captured by the receiver antenna. The resulting image of the subsurface is generated by processing the captured electromagnetic waves. An application of this technology for the remote sensing and determination of the permittivity of materials was proposed by Song [53]. In their research, the authors use the relationship between permittivity and the reflection coefficient to estimate permittivity as follows:

(12) $$\varepsilon ={\left(\frac{1+\gamma }{1-\gamma }\right)}^{2},$$

where the reflection coefficient (γ) is given by the ratio between the amplitude of the reflected signal from the surface ($A_{s}$) and the amplitude of the reflected signal from the medium ($A_{m}$).

(13) $$\gamma =\frac{A_{s}}{A_{m}},$$

*Electrical capacitance volume tomography (ECVT)*: This method consists of electrodes that form a sensor array, arranged around the material or area of interest, which measure and collect the capacitance data between different pairs of electrodes. These data reflect the distribution of dielectric permittivity within the volume of the MUT and are post-processed to reconstruct a three-dimensional image of the material’s dielectric properties [54]. Studies such as those by Muhammad [55] have compiled and compared mathematical models to calculate soil moisture using dielectric measurements with ECVT.

The methods that determine permittivity as listed above, such as TDR, capacitance methods, and VNA, provide foundational techniques for measuring dynamic changes in dielectric properties during copper leaching processes. Each method, with its unique advantages and limitations, is summarized in Table 2. After studying these techniques, the discussion can now focus on how these permittivity measurements can be strategically applied to optimize the leaching process. This includes real-time monitoring to enhance process control and ultimately increase the efficiency and sustainability of copper extraction operations. Integrating advanced sensor technologies into the leaching process not only addresses current technological gaps but also paves the way for future innovations in the field. Also, as shown in Figure 3, material permittivity measurement is highly dependent on the frequency and mode of excitation. Hence, one also needs to consider what are the best ranges of frequencies to be used for the desired excitation mode.

## 4. Dielectric Properties of Copper Minerals

In heap leaching, the heap can act as a complex dielectric medium composed of minerals, solvents, and air gaps. The electrochemical characteristics of the heap, including its electrical resistance and ion migration behavior, are crucial for the efficiency of the leaching process. Variations in the composition and physical state of the heap material can significantly influence these properties. For instance, the pore structure and connectivity within the heap affect the solution resistance (Rs) and charge transfer resistance (Rt), which are critical parameters in the leaching process. Understanding these characteristics is essential for developing monitoring systems and optimizing the leaching process [56].

The dielectric properties, specifically relative permittivity, play a crucial role in how the electric field interacts with the heap during the leaching process. Materials with high permittivity, such as wet ores, can concentrate electric fields, thereby influencing the leaching process. This principle is central to the application of microwave technology in mineral processing, as discussed in the paper by Chen [57]. The paper emphasizes the importance of dielectric properties in microwave heating, where materials with high relative permittivity absorb microwave energy more efficiently, leading to rapid and uniform heating. This characteristic is particularly beneficial in ore pre-treatment and leaching processes. Microwave heating has been demonstrated to improve the recovery of metals from ores by inducing thermal stress and creating micro-fractures within the mineral structure, enhancing leaching efficiency by increasing the surface area exposed to leaching agents. The selective heating capability of microwaves allows for the targeted heating of specific mineral phases, optimizing the leaching process. Literature reviews on copper sulfide pretreatment, such as those by Neira [58], align with the findings of Chen [57], highlighting the advantages of utilizing dielectric properties to enhance metal recovery. However, while the technical benefits of microwave heating are well documented, its economic viability at the operational level remains to be proven. Future research directions include developing more cost-effective microwave systems and integrating microwave technology with conventional processing methods to enhance overall efficiency and reduce costs.

Research by Moravvej [59] aims to demonstrate that electromagnetic irradiation significantly enhances the rate and ultimate recovery of copper from low-grade copper sources. Key findings indicate that microwave treatment can lower H_2_SO_4_ consumption in leaching tests compared to conventional methods. The study involved two types of ores, sulfide and oxide, and showed a significant increase in copper recoveries from pretreated ores, with improvements of up to 26% in some cases. The main effective parameters studied include irradiation power, ore states in the microwave chamber (dry and slurry states), and irradiation time. The results showed that higher microwave power and longer irradiation period positively affected copper recovery. Specifically, microwave irradiation in the slurry state significantly increased copper extraction compared to the dry state, and this can be attributed to the higher dielectric constant and therefore higher degree of polarization. Additionally, the study found that microwave pretreatment led to lower acid consumption, with a reduction of about 28.8% for sulfide ore and 10.5% for oxide ore. In a similar study, but now in the radiowave (RW) range, Moravvej [60] explored the impact of RW on fixed-bed column leaching. Copper recovery increased by about 40% under RW irradiation compared to conventional methods, while agitated slurry leaching showed up to a 10% improvement. The enhanced recovery is attributed to dielectric heating, where electromagnetic waves cause rapid and uniform heating, particularly in minerals with high dielectric constants like copper minerals. RW irradiation also facilitates the diffusion of a lixiviant into the mineral surface by creating capillary cracks, enhancing the leaching process. When analyzing slurries at different densities, the results show that lower densities draw higher recoveries, again attributed to the polarization mechanism. Additionally, RW irradiation reduces leaching time and acid consumption, with higher irradiation power and longer exposure times positively affecting copper recovery.

In Onol [61], the authors draw a comparison between microwave leaching and conventional methods, focusing on parameters such as leaching media, temperature, and time to determine optimal conditions for copper dissolution. Microwave leaching was found to be more efficient, with optimal conditions identified as a solid-to-liquid ratio of 1:100 g/mL, a temperature of 140 °C, a solution of 0.5 M H_2_SO_4_ + 0.05 M Fe_2_(SO_4_)_3_, and a leaching time of 1 h. Increasing the temperature improved copper leaching efficiency for both methods, but microwave leaching achieved higher efficiencies due to its ability to overcome the boiling point limitation of conventional heating. The leaching efficiency increased with time for both methods, with microwave leaching consistently showing higher efficiency. Adding Fe_2_(SO_4_)_3_ as an oxidizing agent significantly increased copper leaching efficiency, especially at higher temperatures, with microwave leaching enhancing copper recovery from 33.37% to 41.37% at 100 °C. The dissolution efficiencies of various ores and concentrates were higher in microwave leaching, with SEM and XRD analyses showing more cracks and smaller particle sizes in microwave-treated samples. The study concludes that microwave leaching provides higher copper leaching efficiencies than conventional methods, suggesting that microwave heating can significantly enhance the efficiency of copper extraction processes.

The authors have used microwave pretreatment or exposure to electromagnetic radiation to enhance recovery rates on leaching copper ores, taking advantage of the dielectric properties of copper minerals. As the leaching ore changes its chemical composition throughout the process, so do its dielectric properties. Assessing the interaction between microwaves and ore under different conditions suggests possible monitoring applications to estimate ore conditions and/or internal structure. At the operational level, this can provide insights into which areas of the ore will leach most effectively and which minerals are most likely to react. The next subsection explores how these dielectric properties can be utilized for the detection of copper.

### Permittivity Measurements in Copper Detection

The pregnant leach solution from copper leaching typically contains a complex mixture of dissolved ions, solids, and leaching agents, such as sulfuric acid. Among these, copper ions (Cu^2+^) could be the primary target for monitoring. The ability to monitor the concentration of copper ions accurately and efficiently in the PLS is crucial for the control and optimization strategies of the leaching process [8,62].

By observing the lack of technical solutions for the detection of the leakage of contaminants into soil, Pashtun [63] incorporated TDR for the detection of water and copper sulfate leakage in copper mine soil. The developed system was tested under different concentrations of the contaminant (in the range of 1 g/L to 5 g/L). The document focuses on the calculation of the dielectric constant of the contaminated soil, as well as its conductivity. The results are shown to be valid, with concentrations of copper sulfate up to 5 g/L, and they indicate that an increase in copper sulfate increases the dielectric permittivity. The experiments demonstrate the causation of changes in the dielectric constant given by the changes in the concentration of the contaminant in the measured material. However, the resulting graphs indicate that there is a nonlinear relationship between the two, showing an abrupt change in the dielectric constant value at around a concentration of 3 g/L.

Similarly, in an effort to detect and conduct the real-time monitoring of copper, Frau [64,65] proposed functionalized microwave sensors that can effectively detect and quantify copper and zinc concentrations in both laboratory-prepared and real-world mine water samples. The sensors demonstrated a strong linear correlation (R^2^ = 0.99) between the reflection coefficient magnitude (S_11_) and the concentration of copper and zinc at 0.44 GHz. One of the main challenges was ensuring the specificity of the sensors for the target contaminants, as the presence of similar toxic metals could potentially interfere with the detection. To address this, the sensors were functionalized with coatings of l-cysteine, chitosan, and bismuth zinc cobalt oxide, which improved specificity by chemically interacting with copper and zinc ions. Another challenge was identifying changes in the resonant frequency peaks related to variations in metal ion content. The study found that functionalized sensors showed higher sensitivity and specificity, particularly at frequencies between 0.92 and 1.00 GHz, compared to uncoated sensors. Field tests on water samples from mining areas demonstrated high repeatability and the ability to differentiate pollution levels, confirming the potential for specific metal detection in complex water matrices. The research highlights the feasibility of using microwave sensors for real-time environmental monitoring, with functionalized sensors providing enhanced sensitivity and specificity. Further research is needed to address interferences from complex water matrices and develop more robust sensor arrays for specific metal detection.

Haryono [66] conducted a study using electrical capacitance volume tomography (ECVT) to monitor the copper leaching process, focusing on mapping the leaching reaction over time. They utilized permittivity measurements, which vary with ion concentration changes due to the leaching reaction, to track the process. The ECVT method, with its high temporal and spatial resolution, allowed for the real-time volumetric imaging of the leaching process. The sensor array, consisting of 24 hexagonal electrodes, was optimized for accurate data collection. The experiment involved copper foil and 2 M H_2_SO_4_ as the leaching agent, with the ECVT system capturing and reconstructing electrical signals into 3D images. The results revealed the heterogeneous nature of the leaching process, with varying permittivity distributions indicating different reaction intensities across regions. Over time, a decrease in permittivity signals was observed, reflecting changes in ion concentration. This study demonstrates ECVT’s potential for providing detailed, real-time insights into the leaching process, highlighting its applicability for industrial-scale monitoring.

Geophysical methods have been applied to heap leaching operations to study the subsurface of a leach pad by Moradipour [67]. In their paper, the authors propose the utilization of techniques such as GPR, electrical resistivity (ER), and induced polarization (IP) as non-destructive methods to map the different levels of acid saturation and concentration of copper minerals in the subsurface of a leach pad. The study was conducted at the *Sarcheshmeh* copper mine in Iran, where these methods were applied to determine acid-saturated zones within heap No. 3. The results indicate that higher saturation correlates with lower resistivity and vice versa, while higher induced polarization values indicate higher concentrations of minerals and acid saturation. Specifically, the electrical resistivity method was used to identify low-moisture and acid-saturated areas by measuring the resistivity values, which ranged from 2 to 13 Ωm. GPR data, collected using a 50 MHz LOZA system, further helped in identifying these zones, although the depth of investigation was limited to about 30 m due to radar wave attenuation in the high-conductivity material. GPR images correlate and show the distribution of the saturated and dry areas of the heap obtained with the other methods. The integration of these geophysical methods, along with laboratory measurements, provided a comprehensive understanding of subsurface conditions, revealing the preferential flow paths of the leaching solutions and ensuring the proper performance of the heap leaching pad. The authors make a case for the integration of different noninvasive methods to estimate the areas and PLS paths for improved operations.

## 5. Discussion

### 5.1. Analysis and Thoughts

The hydrometallurgical extraction of copper has gained significant attention for its sustainability and cost-effectiveness compared to traditional methods. Driven by the growing demand for copper in renewable energy and electric vehicles, this shift necessitates more efficient and environmentally friendly extraction techniques. So far, economic heap leaching focuses on the extraction of copper from oxide ores using sulfuric acid as the main reagent. However, current efforts for the economical heap leaching of copper sulfide ores might require more expensive chemicals, increasing the costs for the overall operation.

To enhance, manage, and standardize the production of PLS in the heap, the development of both first principles and data-driven models has proven to be crucial. These models are essential for accurately estimating recovery rates and implementing effective control strategies in production processes. Currently, there is a strong push towards adopting intelligent strategies that incorporate ML-assisted decision-making and advanced process controls. These advanced methods have demonstrated significant benefits in optimizing production efficiency and effectiveness. In Saldaña [6], the author’s efforts in obtaining stochastic models show promising results using the parameters of the heap composition, such as particle size, porosity, reagent flow, and the height of heap, with some influence on the model’s results. This approach shows that stochastic models are suitable for estimating recoveries. However, the authors do not include other physical parameters such as moisture, pH, or temperature, which are relevant to the first principles’ recovery models and dynamics. This is later addressed by Saldaña [10], where the authors provide a comprehensive selection of mathematical models for copper leaching. In this context, it could be advantageous to build a thermodynamics-informed data-driven model that integrates mathematical modelling with sensor data. However, the success of these approaches heavily relies on the availability and quality of diverse and reliable data. Therefore, sensor technologies play a crucial role in enhancing this process and enabling real-time monitoring.

A different approach, crucial for the development of enhanced leaching technology, involves studying the electrochemical and dielectric properties of leaching ores. This includes microwave and radiowave heating and the pretreatment of leaching chalcopyrite ores. Onol [61] demonstrate an increase in recovery with microwave heating during the leaching process. However, the authors do not directly discuss the operational-level applications of this technology. More recently, Chen [58] discussed the implementation of this technology, suggesting that it could be integrated into mineral processing plants. The paper emphasizes pyrometallurgical aspects and does not delve deeply into hydrometallurgical applications.

Other proponents of utilizing the dielectric properties of copper ores are Batchelor [68] and Ferrary-John [69]. Their studies expand the use of microwave and radiofrequency technologies for ore selection and sorting by analyzing temperature patterns after pretreatment. These experiments, conducted in pilot plants, serve as proof of concept for the technology. Subsequently, Batchelor [70] proposed large-scale implementation, highlighting the benefits of microwave technology while being conservative about power usage and its application to different ore compositions.

The use of microwave technology extends beyond heating mineral samples. Catala-Civera [71] and Garcia-Banos [72] monitor mineral heating by leveraging electromagnetic properties, demonstrating that microwaves can be used for heating, followed by the measurement of the dielectric properties reflected in the permittivity. Similarly, in Duan [73,74] and Mirza [75], the authors detect mineral contents and generate two-dimensional images of core samples using a microwave antenna array and ML sorting algorithms. While these studies show outstanding results, they lightly touch on the large-scale implementation and real-time monitoring of minerals. Furthermore, the research conducted in Frau [64,65] demonstrates that microwave sensors can be functionalized to detect the ionic concentrations of copper and other metals at specific frequencies. Although these sensors are primarily designed for monitoring contaminated groundwater, there is potential to apply these principles to track valuable metal concentrations in PLS or in leach pads. This would necessitate the development of sensors that are resistant to sulfuric acid, research on the correlation between leaching and permittivity, and studies on the scalability and implementation of the technology to monitor the heap.

When discussing operational-level applications, Moradipour [67] set a precedent for the integration and deployment of electromagnetic techniques to assess leach pad conditions. Building on this foundation, there are significant opportunities for the installation of permanent sensors in the leaching field to continuously monitor these conditions. These sensors could provide real-time data on various parameters such as moisture content, temperature, and chemical composition, enabling more precise control and the optimization of the leaching process.

Regarding the correlation between leaching time and permittivity, the findings reported by Haryono [66] indicate that it is possible to monitor the leaching process in the liquid phase through permittivity measurements using ECVT. The scalability of this system relative to industrial applications could be realized downstream of heap leaching, potentially to monitor copper concentrations in the solvent extraction and electrowinning (SX/EW) stages.

The effective monitoring of heap and PLS in copper leaching processes is essential for enhancing both efficiency and sustainability. The integration of advanced sensor technologies has demonstrated significant potential in the real-time detection and quantification of copper and other materials. Furthermore, methods like electrical capacitance volume tomography provide innovative approaches to visualize and comprehend the leaching process, thereby contributing to improved process control and optimization. There remains a need for cost-effective, non-destructive, and real-time monitoring solutions to support optimal reagent irrigation and maximize copper extraction.

### 5.2. Practical Implementation of Permittivity Monitoring

The strategic positioning and distribution of permittivity-based sensors on the leach field are crucial for the effective monitoring and optimization of the leaching process. An approach similar to ore body estimation by core drilling could be implemented, where the spatial distribution of the sensors is recorded, integrated, and merged with ore permittivity parameters from different sensors. Therefore, proper sensor placement can significantly enhance real-time irrigation patterns and enable the precise mapping of target mineral content across areas of the pad, yielding substantial benefits for copper mines. Additionally, placing sensors at different depths as the pad is developed would allow for monitoring vertical variations in permittivity, which can indicate changes in ore composition over time.

It is also essential to locate these sensors to avoid interference with mining equipment and ensure consistent data transmission. Interpreting permittivity data in real-world mining environments requires a robust understanding of the factors influencing measurements. Variations in ore composition, moisture content, and temperature can all impact permittivity readings. Advanced data analysis and integration with other techniques and sensors and developing machine learning algorithms can help identify patterns and correlations in the data. These techniques can be used to develop predictive mapping models that account for the dynamic nature of the leaching process.

Calibration is essential to ensure the accuracy of permittivity measurements. Sensors must be calibrated against known reference materials with well-defined dielectric properties under controlled conditions. Substances such as deionized water, air, and samples of agglomerated ore can provide a baseline for the calibration of dielectric permittivity sensors. Sensor drift must be accounted for; thus, regular calibration checks are also necessary to prevent cumulative errors in data. In situ calibration techniques, such as reference probes placed in the leach pad or non-leached ore, can help maintain accuracy over time. Calibration data should be logged and analyzed to detect deviations that might indicate sensor malfunction or environmental changes affecting measurements.

### 5.3. Integration with Other Sensors

There is significant potential for using radio and microwave dielectric permittivity measurements to detect copper in leaching ore. Integrating these measurements with established techniques such as GPR, IP, and ER can offer complementary benefits, enhancing the reliability and comprehensiveness of monitoring systems. For instance, GPR can be used to map the initial moisture content and composition of the leach pad, providing a baseline for permittivity measurements. It can also validate and refine the spatial distribution of dielectric properties through imaging, offering a more detailed understanding of the leaching process. Similarly, integrating permittivity measurements with IP and ER can enhance the detection of copper and improve the accuracy of monitoring changes in mineral compositions during leaching. By measuring the chargeability of high and low acid-saturated zones, these techniques can help identify mineral-rich areas within the leach pad, providing more precise and reliable data on mineral composition and improving raffinate distributions.

In addition, other monitoring tools, such as pH and temperature probes, can also contribute to a more complete view of the leaching process, enabling more precise control and optimization. The fusion of data from multiple sources allows mining operations to develop more robust models of the leaching process. This multisensory approach can help identify anomalies, reduce measurement uncertainty, and improve the accuracy of predictions. Advanced data-informed dynamical models, including machine learning algorithms, can be used to integrate and analyze data from the different sensors, providing a holistic view of the leaching process.

### 5.4. Opportunities, Advantages, and Technological Gaps

From an automation perspective, it is crucial to recognize that a process variable that cannot be measured is nearly impossible to control. Automating mining processes offers several advantages compared to human-operated processes. Advancements in sensor technology play a key role in achieving this automation. Incorporating permittivity measurements for real-time monitoring and integrating them with process control systems can significantly enhance operational efficiency. Supervisory control and data acquisition (SCADA) systems, such as fuzzy control or model predictive control (MPC) controllers, along with AI algorithms, can dynamically adjust leaching parameters based on real-time data, ensuring that the process remains efficient and effective. This proactive approach minimizes operational downtime, maximizes resource utilization, and improves overall productivity.

Along with the potential benefits, there are technological gaps and challenges associated with using permittivity measurements for monitoring the copper leaching process. When collecting permittivity data directly from the leaching ore, there are several factors that can influence the measurements. The particle size distribution of leached ore undergoes significant changes as sulfuric acid removes valuable minerals such as copper, leading to a gradual reduction in particle size. Also, changes in ore porosity can have an effect on the permittivity of the ore. Additionally, the transport of percolating acid within the ore causes small particles to migrate towards the bottom, actively altering the permeability of the pad. The ionic concentration of the pregnant leach solution exhibits dynamic variations during the leaching process. The dissolution rates of different ions, such as Cu^2+^ and Fe^3+^, into the solution contribute to fluctuations in permittivity measurements. Understanding these interconnected phenomena is essential for accurate permittivity measurements in ore leaching processes. By considering the influence of particle size distribution, percolating acid transport, and dynamic changes in ionic concentration, the copper mining industry can gain valuable insights into the behavior of leaching ores. Implementing permittivity measurements for monitoring the copper leaching process would require a multi-faceted approach:

- *Develop standardized methodologies*: Currently, there is a lack of standardized methodologies for using permittivity measurements in the context of copper leaching. This makes it difficult to compare results across different studies or industrial settings. Standardized methodologies for the measurements in the context of copper leaching need to be developed. This means defining measurement procedures, data analysis methods, and interpretation guidelines.
- *Improve measurement accuracy*: The copper leaching process involves various parameters such as ore composition, acid concentration, solid-to-liquid ratio, particle size, and temperature. These factors can influence the permittivity of the materials involved, adding complexity to the interpretation of permittivity measurements. This variability can lead to significant measurement uncertainty, necessitating efforts to increase accuracy. Implementing a multisensory approach along with state estimation algorithms (e.g., soft sensors, Kalman filters, regression models) can help mitigate these challenges.
- *Integrate with existing process monitoring tools*: Permittivity measurements should be integrated with existing process monitoring tools to provide a comprehensive view of the leaching process. This would allow for a more accurate and holistic monitoring of the process.
- *Conduct further research*: Further research is needed to better understand the relationship between permittivity and the various parameters of the copper leaching process. Experimental studies, as well as the development of mathematical models, are necessary for the success of this technology.

While there are challenges associated with the implementation of permittivity-based sensors, the benefits they offer can significantly outweigh them, making these sensors valuable instruments for monitoring and optimizing copper leaching processes. Permittivity-based sensors can provide real-time data on changes in moisture content, ore composition, and ionic concentration dynamically, as opposed to static geotechnical data. Thus, when deployed, more precise control of the leaching operations can be achieved, allowing operators to make informed decisions and adjustments on the fly. This leads to higher efficiency, reduced reagent consumption, optimized leaching conditions, and ultimately enhanced copper recovery rates. By providing detailed insights into the leaching process, permittivity-based sensors help identify areas where acid addition rates can be improved. They can detect zones with suboptimal moisture levels or uneven reagent distribution, allowing for targeted interventions that reduce resource waste, minimize environmental impact, and support more sustainable mining practices. Additionally, optimizing reagent use can lower operational costs while reducing the environmental footprint of mining operations.

Due to the nature of the measurement, permittivity-based sensors are cost-effective in terms of both data processing and equipment. The sensors generate data that can be processed at a lower cost compared to traditional geotechnical methods. Furthermore, equipment such as VNAs is relatively inexpensive, making it accessible for a wide range of mining operations. The ability to deploy multiple probes permanently across the leach pad allows for extensive coverage and monitoring. As the leach pad grows, these sensors can be permanently installed, providing continuous data collection vertically over the lifespan of the operation. When combined with other established techniques, permittivity-based sensors can offer a comprehensive monitoring solution, providing a more detailed understanding of subsurface conditions. Additionally, permittivity-based sensors can be integrated with SCADA systems, allowing for centralized monitoring and control. This integration enables real-time data visualization, automated alarms, and streamlined decision-making processes. SCADA systems can leverage data from permittivity sensors and complementary sensors to optimize leaching operations, improve safety, and reduce waste.

## 6. Conclusions

The paper provides an in-depth review of dielectric permittivity measurements in copper leaching, emphasizing their potential to enhance process control, efficiency, and environmental sustainability. It discusses various techniques, including time-domain reflectometry, capacitance methods, and vector network analyzers, highlighting their respective advantages and limitations.

Permittivity-based sensors present significant opportunities for the real-time monitoring of critical parameters, such as moisture content and mineral composition. This capability can lead to more precise control of the leaching process, improved efficiency, and higher ore recovery rates. Integrating these measurements with existing systems can also facilitate advanced control strategies, leveraging AI and machine learning to dynamically adjust leaching parameters for optimal performance. However, several challenges must be addressed. Several factors such as moisture, temperature, and ionic concentration comprise permittivity measurements, introducing variability and complexity in data interpretation. Additionally, changes in ore properties during leaching, such as porosity and particle size distribution, can impact measurements as well. The lack of standardized methodologies further complicates the comparison of results across different studies and industrial settings.

Future work should focus on developing standardized methods for permittivity measurements, enhancing measurement accuracy, and integrating these measurements with current monitoring tools. Further research is needed to explore the relationship between permittivity and various leaching parameters through experimental studies and mathematical modeling. By addressing these areas, the mining industry can leverage permittivity measurements to significantly improve the efficiency, sustainability, and profitability of copper leaching operations.

## Figures and Tables

**Table 1:** Relative permittivity of various substances.

Mol. Formula	Name	*ε_r_*
Cu_2_O	Cuprous oxide (cuprite)	7.60 ± 0.06 ^1^
CuFeS_2_	Cuprous sulfide (chalcopyrite)	3.176
H_2_O	Water	78.36 ^1^
CaO	Calcium oxide	11.8 ^1^
CaCO_3_	Calcium carbonate	8.67 ^1^
Fe_3_O_4_	Iron (II,III) oxide (magnetite)	20 ^1^
NaCl	Sodium chloride (Salt)	5.9 ^1^
FeS_2_	Pyrite	10.9 ± 0.5 ^2^

^1^ Data obtained from [28]; ^2^ data obtained from [29].

**Table 2:** Permittivity measurement methods, applications, and advantages and disadvantages.

Method	Applications	Advantages	Disadvantages
Time Domain Reflectometry (TDR)	Soil moisture	High accuracy, non-destructive	Requires calibration, expensive equipment
Capacitance Method	Soil moisture	Simple, cost-effective	Affected by temperature and salinity
Network Analyzer	Ionic solutions	High precision, wide frequency range	Complex setup
Resonant Cavity Method	Ionic solutions	High sensitivity, accurate	Limited to specific frequencies
Ground Penetrating Radar (GPR)	Soil moisture	Non-invasive, provides spatial distribution	Limited to specific frequencies
Electrical Capacitance Volume Tomography (ECVT)	Soil moisture	Provides 3D imaging, non-destructive	Complex calibration, expensive

## Data Availability

The data presented in this study are available upon request from the corresponding author.
